# Prognostic factors and survival of patients with brain metastasis from breast cancer who underwent craniotomy

**DOI:** 10.1002/cam4.439

**Published:** 2015-03-09

**Authors:** José Pablo Leone, Adrian V Lee, Adam M Brufsky

**Affiliations:** Division of Hematology and Oncology, University of Pittsburgh Cancer Institute5150 Centre Avenue, Pittsburgh, Pennsylvania, 15232

**Keywords:** Brain metastasis, breast cancer, craniotomy, prognostic factors, survival

## Abstract

Brain metastasis (BM) in patients with breast cancer is a catastrophic event that results in poor prognosis. Identification of prognostic factors associated with breast cancer brain metastases (BCBM) could help to identify patients at risk. The aim of this study was to assess clinical characteristics, prognostic factors, and survival of patients with BCBM who had craniotomy and resection in a series of patients treated with modern multimodality therapy. We analyzed 42 patients with BCBM who underwent resection. Patients were diagnosed with breast cancer between April 1994 and May 2010. Cox proportional hazards regression was selected to describe factors associated with time to BM, survival from the date of first recurrence, and overall survival (OS). Median age was 51 years (range 24–74). Median follow-up was 4.2 years (range 0.6–18.5). The proportion of the biological subtypes of breast cancer was ER+/HER2− 25%, ER+/HER2+ 15%, ER-/HER2+ 30%, and ER-/HER2− 30%. Median OS from the date of primary diagnosis was 5.74 years. Median survival after diagnosis of BM was 1.33 years. In multivariate Cox regression analyses, stage was the only factor associated with shorter time to the development of BM (*P* = 0.033), whereas age was the only factor associated with survival from the date of recurrence (*P* = 0.027) and with OS (*P* = 0.037). Stage at primary diagnosis correlated with shorter time to the development of BM, while age at diagnosis was associated with shorter survival in BCBM. None of the other clinical factors had influence on survival.

## Introduction

Breast cancer is the second most frequent cause of brain metastasis (BM) after lung cancer, with metastases occurring in 10–16% of patients 1. However, autopsy studies have revealed a further 10%, which were clinically nonapparent 2. In recent years, the incidence of BM seem to have increased, probably due to prolonged survival of patients secondary to more efficient treatments and to increased detection of brain lesions with improved imaging techniques.

Previous reports have identified the subsets of patients with triple-negative or human epidermal growth factor receptor 2 (HER2)-positive tumors as having an increased risk for the development of metastatic disease to the brain 3–6. BM in patients with breast cancer is a catastrophic event that results in poor prognosis, with a median survival of 2–9 months despite treatment 7–10. Surgical resection, whole-brain radiotherapy (WBRT), stereotactic radiosurgery (SRS), chemotherapy, and targeted therapy may improve outcomes in these patients 9,11,12. Identification of prognostic factors associated with breast cancer brain metastases (BCBM) could help to identify patients at risk.

The aim of this study was to assess clinical characteristics, prognostic factors, and survival of patients with BCBM who had craniotomy and resection in a series of patients treated with modern multimodality therapy.

## Material and Methods

### Clinical data

We identified 53 patients who underwent craniotomy for metastasis of breast cancer at the University of Pittsburgh Medical Center. Of those, we were able to retrieve clinical information on 42 patients, which were included in this analysis. Patients with leptomeningeal disease or dural metastases without parenchymal brain metastatic lesions were excluded from the study. Patients were diagnosed with breast cancer between April 1994 and May 2010. Information collected included age at diagnosis, demographic data, menopausal status, date of diagnosis, tumor histology and grade, stage at diagnosis, estrogen receptor (ER), progesterone receptor (PR) and HER2 status, dates and types of all treatments – including chemotherapy, hormonal therapies and targeted agents –, response to each treatment, survival status, date and location of distant metastasis, number of BM, and date of last follow-up or death. Staging was documented according to the criteria of the Seventh American Joint Committee on Cancer (AJCC) 13. This study was approved by the University of Pittsburgh Institutional Review Board.

### Statistical analysis

Time to BM was defined as the interval from initial breast cancer diagnosis until the development of BM 7,14. Disease-free survival (DFS) was calculated as the time from first treatment of breast cancer until the date of recurrence 12,15. Survival from the date of first recurrence was defined as the interval from the date of recurrence until the date of death from any cause or last follow-up for patients that were censored. Overall survival (OS) was estimated as the time from diagnosis of breast cancer until death from any cause or last follow-up for patients that were censored.

Survival probabilities were analyzed using the Kaplan–Meier method. Cox proportional hazards regression was selected to describe factors associated with time to BM, survival from the date of first recurrence, and OS.

Due to our sample size and in an attempt to minimize false-positive results, we determined to study five clinical prognostic factors prior to conducting any analysis: age at diagnosis, stage at diagnosis, receptor status, type of radiation therapy administered to the brain, and type of first rumor recurrence. Age was analyzed as a continuous variable, the rest of the prognostic factors were considered categorical. Alpha level of 0.05 was the cutoff of significance for all test statistics. Analysis was conducted using SAS 9.3(SAS Institute Inc, Cary, NC, USA).

## Results

### Patient characteristics

A total of 42 patients were included in the analysis. Median age at initial diagnosis of breast cancer was 51.65 years (range: 24.85–74.78 years). Patient characteristics are included in Table1. The majority of patients had stage II at diagnosis (40.5%), although patients of all stages were included; most patients were histologic grade 3 (61.9%); ER, PR, and HER2 were predominantly negative (57.1%, 76.2%, and 52.4%, respectively); approximately two-thirds of patients received radiation therapy to the brain and the distribution of the type of radiation was similar between gamma knife, whole-brain radiation therapy, and the combination of the two. A total of 25 of 28 patients (89.3%) received radiation therapy after craniotomy, whereas two patients received radiation before and after surgery and one patient had radiation prior to surgery. Interestingly, brain was the most frequent site of first recurrence (35.7% of cases) and 57.1% of patients relapsed first with either brain or visceral disease.

**Table 1:** Patient characteristics

	*N*	%
Stage		
1	5	11.9
2	17	40.5
3	11	26.2
4	7	16.7
Unknown	2	4.8
Stage subtype		
1A	5	11.9
2	1	2.4
2A	11	26.2
2B	5	11.9
3A	4	9.5
3B	4	9.5
3C	3	7.1
4	7	16.7
Unknown	2	4.8
Grade		
1	1	2.4
2	5	11.9
3	26	61.9
Unknown	10	23.8
ER		
Negative	24	57.1
Positive	18	42.9
PR		
Negative	32	76.2
Positive	10	23.8
HER2		
Negative	22	52.4
Positive	18	42.9
Unknown	2	4.8
Radiation therapy		
No	14	33.3
Yes	28	66.7
Radiation type		
Gamma knife	10	35.7
Whole brain	7	25
Gamma knife and whole brain	11	39.3
Type of recurrence		
Lymph nodes	3	7.1
Bone	7	16.7
Brain	15	35.7
Local	4	9.5
Soft tissue	2	4.8
Visceral	9	21.4
Unknown	2	4.8
Vital status		
Alive	15	35.7
Dead	27	64.3

ER, estrogen receptor; PR, progesterone receptor; HER2 epidermal growth factor receptor 2.

Patients had a median of 2 BM at the time of imaging diagnosis (range: 1–15), the median size of the largest BM was 3.25 cm (range: 0.6–5.9 cm) and they received a median of three lines of chemotherapy (range: 0–11).

The distribution of different biological subtypes of breast cancer was as follows: ER+/HER2− 25%, ER+/HER2+ 15%, ER-/HER2+ 30%, and ER-/HER2− 30%.

### Survival analysis

**Figure 1:**
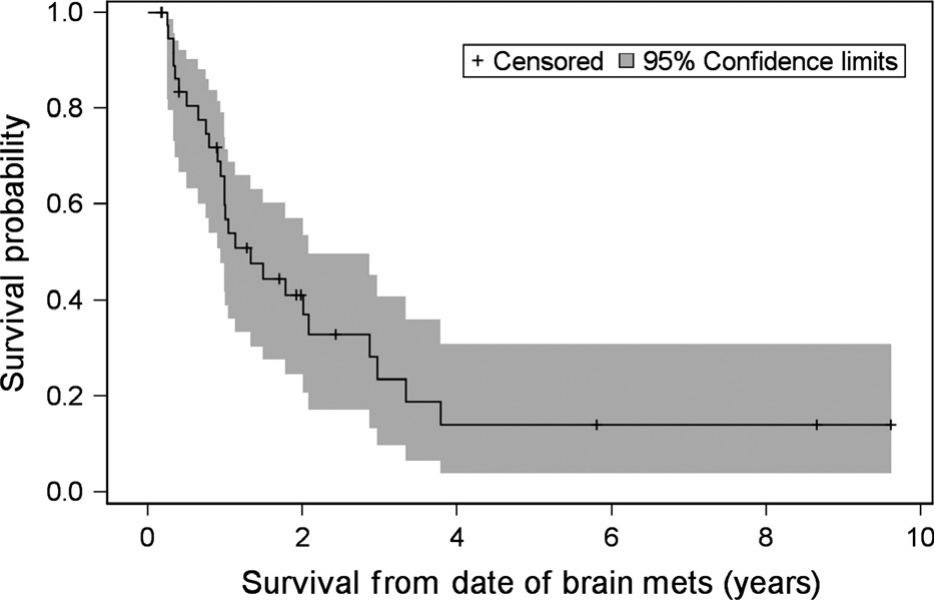
Kaplan–Meier curve for survival from date of brain metastasis.

**Figure 2:**
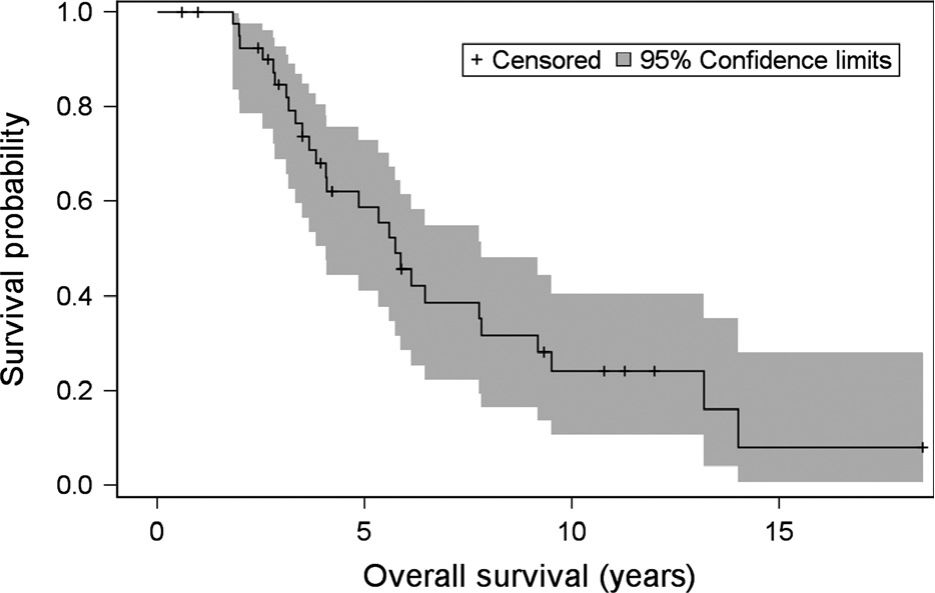
Kaplan–Meier curve for overall survival.

After a median follow-up time of 4.20 years (range: 7.1 months–18.5 years), 15/42 (35.7%) patients are still alive. Median DFS was 2.45 years (95% CI: 1.56 years, 2.89 years). The median time from the diagnosis of breast cancer until the development of BM was 32.24 months (range: 0–206.22 months). After diagnosis of BM, patients had a median survival of 1.33 years (95% CI: 0.95 years, 2.08 years) (Fig.1). Finally, median OS was 5.74 years (95% CI: 4.06 years, 7.81 years) (Fig.2).

A multivariate Cox regression model was conducted to analyze independent predictors associated with time to the development of BM (Table2) and showed that patients who presented with stage 3 or 4 disease had the shortest time to the event, whereas age at the time of diagnosis and the receptor status in the primary tumor had no impact on this endpoint.

**Table 2:** Multivariate model for time to brain metastasis

Factor	Comparison group	Parameter estimate	Standard error	Chi-square	*P*-value
Age		0.005	0.016	0.101	0.751
Stage (ref = stage 1)	Stage 2	0.441	0.593	0.554	0.457
	Stage 3	1.321	0.619	4.549	0.033
	Stage 4	1.356	0.683	3.939	0.047
ER (ref = negative)	Positive	0.397	0.4	0.986	0.321
PR (ref = negative)	Positive	0.32	0.487	0.43	0.512
HER2 (ref = negative)	Positive	−0.423	0.376	1.264	0.261

ER, estrogen receptor; PR, progesterone receptor; HER2 epidermal growth factor receptor 2.

Similarly, age at the time of diagnosis was the only factor associated with both, survival from date of first relapse and OS (Tables3 and 4). Older patients had worse prognosis, they experienced shorter survival after relapse and also shorter OS. Interestingly, stage at initial diagnosis, receptor status in the primary tumor, site of first recurrence, and type of radiation therapy to the brain had no role in predicting survival.

**Table 3:** Multivariate model for survival from the date of first recurrence

Factor	Comparison group	Parameter estimate	Standard error	Chi-square	*P*-value
AGE		0.045	0.021	4.89	0.027
STAGE (ref = stage 1)	Stage 2	0.211	0.638	0.11	0.74
	Stage 3	0.52	0.66	0.619	0.431
	Stage 4	0.879	0.792	1.232	0.267
ER (ref = negative)	Positive	−0.109	0.432	0.064	0.8
PR (ref = negative)	Positive	0.401	0.573	0.491	0.484
HER2 (ref = negative)	Positive	−0.528	0.436	1.466	0.226
Site (ref = brain)	Other	−0.38	0.441	0.743	0.389

ER, estrogen receptor; PR, progesterone receptor; HER2 epidermal growth factor receptor 2.

**Table 4:** Multivariate model for overall survival

Factor	Comparison group	Parameter estimate	Standard error	Chi-square	*P*-value
Age		0.04	0.019	4.359	0.037
Stage (ref = stage 1)	Stage 2	0.277	0.64	0.187	0.665
	Stage 3	1.171	0.714	2.684	0.101
	Stage 4	1.364	0.808	2.852	0.091
ER (ref = negative)	Positive	0.024	0.45	0.003	0.958
PR (ref = negative)	Positive	0.353	0.586	0.363	0.547
HER2 (ref = negative)	Positive	−0.537	0.431	1.55	0.212
Type of radiation (ref = no radiation)	Gamma knife	−0.793	0.627	1.599	0.206
	Whole brain	−0.271	0.729	0.138	0.71
	Gamma knife + whole brain	−0.618	0.598	1.067	0.302

ER, estrogen receptor; PR, progesterone receptor; HER2 epidermal growth factor receptor 2.

## Discussion

BM represents the fourth most common metastatic site for patients with breast cancer and has been associated with worse survival and quality of life 16. The advances in systemic therapy have improved outcomes for patients with metastatic breast cancer; however, the incidence of BM in these patients has increased 17, and there is a need to improve local cancer control in the brain.

Our study analyzed the clinical characteristics and predictors of outcome in patients who underwent craniotomy and resection of BM from breast cancer. The median age at breast cancer diagnosis in our cohort was 51.65 years. We observed a higher incidence of HER2 overexpression with 45% of cases being positive for HER2 compared with the 20% incidence of HER2 positivity seen in all patients with breast cancer, this is in agreement with previous reports which have analyzed HER2 as a risk factor for the development of BM 18,19. In a similar way, we also observed a lower incidence of luminal A tumors with only 25% of cases, and a 30% incidence of triple-negative disease. According to our results, both HER2-positive and triple-negative patients are at increased risk for development of BM at some point in their course of the disease, and this is an important consideration when assessing individual patient’s risks for relapse into the brain.

A study conducted by Wronski et al. analyzing the role of surgery in the management of BCBM showed that patients who had positive hormone receptors experienced a significantly longer median survival compared with those who had negative hormone receptors (21.9 vs. 12.5 months, *P* = 0.04) 20. Forty percent of patients, however, had unknown hormone receptors status. Our study did not show an independent predictor role neither of the hormone receptors nor the HER2 receptor status on any of the outcomes we analyzed. Several previous reports have also failed to show an association between receptor status and survival 9,12,14,15,21–26.

The majority of our patients (35.7%) had their first distant recurrence into the brain, and the second most common site of first recurrence was other visceral organs (21.4%). This means that more than half of our patients had a form of visceral recurrence at the time of first relapse and this underscores the high-risk nature of our patient population. Interestingly though, 35.7% of patients are still alive.

The median time from initial breast cancer diagnosis to the development of BM was 32.24 months. This is consistent with previous studies 9,27, Lee et al. reported a median interval between primary diagnosis and BM of 32.3 months. Our cohort revealed that patients who presented with stage 3 or 4 disease at the time of initial diagnosis had the shortest interval to BM. In contrast to previous reports 9, younger age was not associated with this event. Despite the high incidence of HER2-positive and triple-negative patients, receptor status did not affect time interval between initial diagnosis and occurrence of BM. After diagnosis of BM, patients survived a median of 1.3 years, which is significantly longer compared to the 2- to 9-month survival reported by other authors 7–10. This difference in survival could be explained, in part, by the fact that our study included modern multimodality therapy consisting of surgery, radiation therapy with WBRT, and/or SRS and chemotherapy in an era of active biologic agents.

The median OS in our cohort was 5.74 years. Older age at the time of initial diagnosis was the only factor that predicted shorter OS and also the only factor associated with shorter survival from the date of first relapse. This is consistent with previous studies which described worse prognosis for older patients with BM 5,21,22,25,26,28; however, this is the first study to identify the impact of age as a prognostic factor in a cohort of patients uniformly treated with craniotomy in an era of contemporary radiation therapies. The major explanation for this finding may be that older patients tend to have poorer physical condition, other associated comorbidities and lower tolerability for systemic treatment. It is important to mention that according to our results, whether the patients received radiation therapy, the type of radiation administered, the stage at initial diagnosis, the tumor receptor status, and the site of first relapse had no association with survival.

We acknowledge that our study has some limitations. It is a retrospective analysis with a small sample size, which limited a potential assessment of a larger number of prognostic factors. We did not analyze patients who did not have craniotomy, which might have introduced selection bias toward patients with fewer comorbidities, less systemic disease, or BM in surgically favorable locations. However, despite these limitations, our study has a number of advantages. The fact that all our patients underwent craniotomy allowed us to conduct a more precise analysis without the potential confounder of whether surgery was part of the therapy. It is a single institution analysis where patients received similar treatment and follow-up. Finally, by selecting only five prognostic factors prior to the analysis of the data, we minimized potential false-positive results and confirmed the independent validation with the multivariate model.

It is important to mention that our study analyzed patients over a period of 16 years, which provided mature follow-up. However, treatment patterns, radiation modalities, and staging criteria have changed during that time. To address this and minimize potential confounding factors, we used the AJCC breast cancer staging seventh edition 13, patients received treatment according to the standard of care at the time of diagnosis and all treatments were documented and analyzed. For the possible changes in radiation modalities, we used the group of patients who had no radiation as the reference group in the multivariate model, which allowed us to discern between different radiation techniques.

In conclusion, our results showed that patients who presented with stage III or IV disease at initial diagnosis had a significantly shorter time to the development of BM. Age at initial diagnosis was associated with survival from the date of first recurrence and with OS. Notably, neither the biological subtype of cancer, the radiation modality nor the site of first recurrence showed any impact on survival in this subset of patients.
